# 

**Published:** 1905-04

**Authors:** 


					﻿uwentietb Ji?ear.
Vol. XX.
APRIL, 1905.
No. 10. ‘
TEXAS
Medical Journal.
Subscription, $i.oo a Year, in Advance.
’Single Copies, io Cents.
PUBLISHED AT AU8TIM, TEXAS. BT
F. E. DANIEL, M. D.,
Proprietor.
__ PHtlgrgnlRV the VON BOEOKMAN5WONE8 CO.
Entered at the Postoffice at Austin, Texas, as Second-class Mail Matter.
				

